# Changes in Health-related Quality of Life Among Impoverished Persons in the Free/Low-Cost Medical Care Program in Japan: Evidence From a Prospective Cohort Study

**DOI:** 10.2188/jea.JE20210005

**Published:** 2022-11-05

**Authors:** Daisuke Nishioka, Chisato Tamaki, Noriko Furuita, Hirokazu Nakagawa, Erin Sasaki, Rika Uematsu, Takeshi Ozaki, Satoshi Wakata, Naoki Kondo

**Affiliations:** 1 Department of Medical Statistics, Research & Development Center, Osaka Medical and Pharmaceutical University, Osaka, Japan; 2 Department of Health and Social Behavior, Graduate School of Medicine, The University of Tokyo, Tokyo, Japan; 3 Department of Social Epidemiology, Graduate School of Medicine, Kyoto University, Kyoto, Japan; 4 Kyoto Kyoritsu Hospital, Kyoto, Japan; 5 Kyoto Min-iren Chuo Hospital, Kyoto, Japan; 6 Department of Preventive Services, Kyoto University School of Public Health, Kyoto, Japan; 7 Kyoto Min-iren, Kyoto, Japan; 8 Kamigyo Clinic, Kyoto, Japan; 9 Institute for Future Initiatives, the University of Tokyo, Tokyo, Japan; 10 Japan Agency for Gerontological Evaluation Study (JAGES Agency), Tokyo, Japan

**Keywords:** poverty, healthcare access, Free/Low-Cost Medical Care Program, social welfare, Japan

## Abstract

**Background:**

The Free/Low-Cost Medical Care Program (FLCMC) can subsidize the payment (exempt/lower) in designated institutions in Japan. Given that poverty is a multidimensional concept including social isolation, the FLCMC applicants may need social support over and above financial aid to improve their quality of life. However, there was no data to discuss what services should be provided and to whom. Hence, we aimed to describe the changes in health-related quality of life scores among users of the FLCMC, with respect to their socioeconomic backgrounds.

**Methods:**

This cohort study included patients who newly used FLCMC from July 2018 to April 2019. We used patients’ social work records, obtained at baseline, and self-report questionnaires on the Medical Outcomes Study 8 Items Short Form Health Survey (SF-8), measured both at baseline and 6 months after the application. We used the change in physical and mental health component summary scores (PCS-8 and MCS-8, respectively) as outcome variables.

**Results:**

Multiple linear regression analyses, adjusting for age, sex, healthcare institute, and baseline PCS-8 and MCS-8, showed that lower income was associated with an increase in PCS-8 (coef. −0.09; 95% CI, −0.15 to, −0.03) and MCS-8 (coef. −0.04; 95% CI, −0.11, to 0.03). Living alone (versus living with someone) was potentially associated with a decrease in both PCS-8 (coef. −1.58; 95% CI, −7.26 to 4.09) and MCS-8 (coef. −3.62; 95% CI, −9.19 to 1.95).

**Conclusion:**

Among patients using FLCMC, those who live alone may need additional support. Further study testing the generalizability of the findings is required.

## INTRODUCTION

People in financial poverty face barriers to healthcare access despite their medical needs.^1^^–^^3^ Maintaining and advocating for health in the impoverished population is a societal concern; thus, governments in many countries have ensured minimum income as well as financial healthcare access with social assistance entitlement for the poor.^4^ The Japanese government likewise provides a public assistance program that ensures recipients’ monthly minimum income protection and exempts payments for their medical care utilization.^5^ However, the implementation of public assistance programs requires several assessments in terms of ability to work and income, as well as the assets of the applicants and their families, which resulted in time for the decision of the application.

In Japan, the Free/Low-Cost Medical Care Program (FLCMC) is one of the support mechanisms for people in financial poverty and in need of medical care; it is governed by the Social Welfare Act.^6^ The purpose of the FLCMC is to provide “free or low-cost medical care to people with financial difficulties so that they are not restricted in their access to necessary medical care for financial reasons”.^6^ Designated healthcare institutions can apply for the program; subsequently, patients can receive healthcare services at lower or no cost if they meet the individual institutions’ unique eligibility criteria. In 2018, the FLCMC was offered in 673 medical facilities to approximately 7.5 million beneficiaries in total, and has been growing in recent years, partly owing to the rising impoverished older population.^7^

Owing to the existence of the formal public assistance program, the importance of the FLCMC has been questioned in policy debates, and data for policy discussions are lacking.^6^ Recent cross-sectional studies have revealed that FLCMC applicants have low educational attainment, poor health-related quality of life (HRQOL), and limited social interaction.^8^^,^^9^ Given that poverty is a multidimensional concept including non-financial difficulties, such as social isolation and exclusion from communities,^10^ the applicants may also have other difficulties besides financial burden.^8^^,^^9^

The free/reduced-cost care program in the United States, which is aimed at patients who need assistance with medical payments but are not enrolled in Medicaid, is similar to the FLCMC.^11^ The free/reduced-cost care program has been implemented as a charitable initiative in private hospitals. Recipients can receive both financial assistance and social support, contributing to health benefits.^12^ However, the FLCMC differs from this program in that it only provides financial support. Depending on social background, social support may also be necessary to improve quality of life, but there has been no study describing the association between social background and health-related outcomes among FLCMC recipients.

Hence, we established a registry of FLCMC users at two healthcare institutions in Kyoto, based on which we sought to identify the association between socioeconomic factors and changes in FLCMC applicants’ HRQOL.

## METHODS

### Study design, setting, and participants

**Figure 1.**
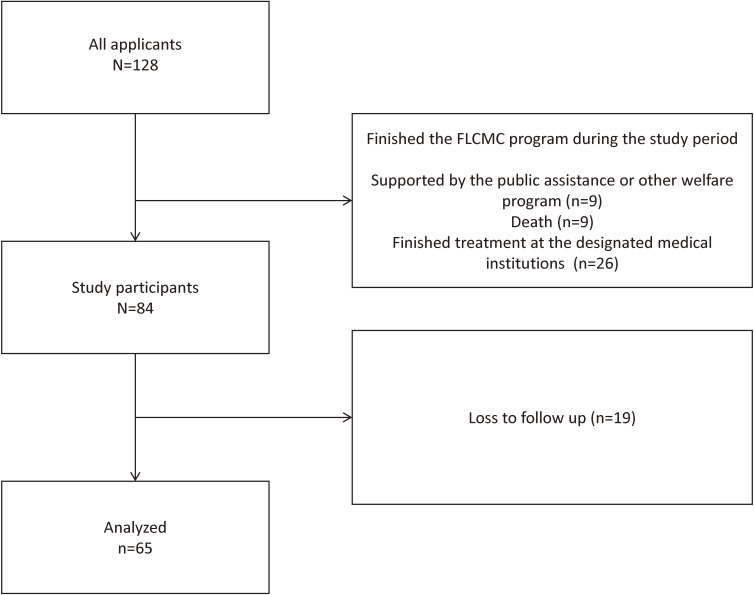
Study flow diagram. FLCMC, Free/Low-Cost Medical Care Program.

This prospective cohort study used data from the FLCMC patient registry that we created at two healthcare institutions in Kyoto city. These well-known institutions have offered the FLCMC since its inception and have also compiled the book on the FLCMC in Japan.^13^ We registered new recipients of the FLCMC aged 20 years or older from July 2018 to April 2019. Registered patients were observed for 6 months from the beginning of their use of FLCMC benefits. We chose the period because 6 months was enough for patients to use formal welfare programs (eg public assistance), we could evaluate before-after change of people continuously on the FLCMC. Then, we excluded patients who were no longer recipients of the FLCMC during the 6 months of the study, which could be because of receiving public assistance or benefits from other welfare programs, death, or completion of treatments at the designated healthcare institutions. Treatments at healthcare institutions were considered complete with the cure of patients’ acute diseases, referral to non-FLCMC-designated institutions, or admittance in nursing homes or long-term care facilities (Figure 1).

### The FLCMC

The FLCMC program is provided through cooperation between designated healthcare institutions, community social welfare councils, and municipal welfare offices.^14^ These institutions agree on patients’ eligibility for the FLCMC based on household income. FLCMC applicants are exempted from payment at the designated institutions, which then cover their medical care costs. Designated institutions can reap the benefits of tax exemptions according to the proportion of patients who use the FLCMC program.^14^

In the healthcare institutions participating in the present study, medical social workers (MSWs) interviewed prospective patients with financial difficulties and determined their eligibility for the FLCMC. The FLCMC eligibility criterion in the two institutions was having a household income of less than 150% of the poverty line, based on which households are eligible for public assistance. Eligible patients can be exempted from all out-of-pocket expenses at the designated healthcare institutions.

### Data sources

The MSWs interviewed patients and recorded their socioeconomic attributes, such as number of household members and household income; this information was required to determine their eligibility for the FLCMC. Moreover, the MSWs described the FLCMC study registry and asked patients if they wished to participate. If patients agreed to participate, the MSWs asked them to answer the additional self-report questionnaires on educational attainment, social interaction, and HRQOL using the Japanese version of the Medical Outcomes Study 8-Item Short Form Health Survey (SF-8).^15^ These questionnaires were to be completed twice—at baseline (during FLCMC application) and after 6 months.

### Variables

#### Outcome variables

We used the 6-month change in HRQOL scores. The SF-8 comprises eight subscales. In this study, scores on the physical health component summary (PCS-8) and mental health component summary (MCS-8) were utilized based on the scoring method used in a large-scale population study in Japan.^11^ We calculated the before-and-after change in both PCS-8 and MCS-8 scores.

#### Explanatory variables

We used data on household composition (living alone or not), educational attainment (<10 years, 10–12 years, or >12 years), and monthly household equivalent income (continuous) as patients’ socioeconomic attributes.

#### Covariates

We used data on age (continuous), sex (male or female), and healthcare institutions. We coded each healthcare institution as a dummy variable to adjust for the unmeasured characteristics of the two institutions (A or B). Further, we used baseline HRQOL scores to control for possible floor and ceiling effects.

### Statistical analysis

First, we described the participants’ baseline characteristics. We summarized the mean and standard deviation (SD) of PCS-8 and MCS-8 scores for both baseline and follow-up. Second, we employed univariate linear regression to calculate the crude coefficient representing the change in HRQOL score and the 95% confidence interval (CI) for the unit differences in each explanatory variable. We then performed multiple linear regression analysis, adjusting for age, sex, healthcare institution, and baseline PCS-8 and MCS-8 scores, to calculate the multivariable-adjusted coefficients of the changes in HRQOL scores and the 95% CIs of each explanatory variable. The robust standard error estimator was adopted to calculate 95% CIs. Further, we performed sensitivity analyses to consider potential high correlations between each variable. We calculated correlation coefficients between each variable and employed multiple linear regression excluding the variables that showed high correlations. Owing to our small sample size, we conducted post-hoc power analysis. All analyses were performed using STATA SE V.16.2 (Stata Corp, College Station, TX, USA).

### Ethical considerations

The participants were informed by the MSWs in charge that their decision on whether to participate in this registry did not affect their application for the FLCMC. The study protocol was approved by the Ethics Committee of the Graduate School of Medicine of the University of Tokyo (Approval No: 11995) and the Ethics Committee of Kyoto Min-iren Chuo Hospital (Approval No: 94). All participants provided written informed consent.

## RESULTS

We obtained data on 128 individuals. Among them, 44 finished their application for the FLCMC during the observational period, and 84 were found to be eligible during this study. As 19 patients were lost to follow-up, the data of 65 patients were analyzed (Figure 1). The sample included 34 males (52.3%), 23 patients living alone (35.4%), and 33 patients with low interpersonal exchange (50.8%). The baseline mean score of the PCS-8 was 39.9 (SD, 8.7) and that of the MCS-8 was 42.8 (SD, 9.1) (Table 1). The follow-up mean score on the PCS-8 was 38.9 (SD, 9.9) and that on the MCS-8 was 43.1 (SD, 8.3).

**Table 1.** Baseline characteristics of patients eligible for the Free/Low-Cost Medical Care Program (*N* = 65)

Characteristic	Category	*n* (Mean)	% (SD)
Age, years		(67.6)	(13.7)
Monthly household equivalent income, JPY		(98,388.4)	(34,734.6)
Sex	Male	34	52.3%
	Female	30	46.2%
Living alone	Yes	23	35.4%
	No	42	64.6%
Educational attainment, years	≤9	21	32.3%
	10–12	29	44.6%
	≥13	15	23.1%
Medical institution	A	51	78.5%
	B	14	21.5%
Health-related quality of life score	PCS-8	(39.9)	(8.7)
	MCS-8	(42.8)	(9.1)

MCS, mental health component summary; PCS, physical health component summary; SD, standard deviation.

The univariate linear regression analysis showed that income was inversely associated with changes in PCS-8 score (coef. −0.09, 95% CI, −0.16 to −0.02) (Table 2), and its intercept was 7.67. The results of multiple linear regression showed that an increase in income by 1,000 Japanese yen (JPY) was associated with a decrease in PCS-8 and MCS-8 score by −0.09 (95% CI, −0.15 to −0.03) and −0.04 (95% CI, −0.11 to 0.03), respectively. Living alone showed minor associations with a decrease in scores on the PCS-8 (coef. −1.58; 95% CI −7.26 to 4.09) as well as the MCS-8 (coef. −3.62; 95% CI −9.19 to 1.95) when compared to living with someone (Table 2).

**Table 2.** Crude and adjusted coefficients and 95% confidence intervals for changes in PCS-8 and MCS-8 scores by individual characteristics

Characteristic	Category	PCS score		MCS score	
		crude coef. (95% CI)	Adjusted coef. (95% CI)	crude coef. (95% CI)	Adjusted coef. (95% CI)
*Explanatory variables*					
Monthly household equivalent income	by 1,000 yen	−0.09 (−0.16, −0.02)	−0.09 (−0.15, −0.03)	0.01 (−0.06, 0.08)	−0.04 (−0.11, 0.03)
Living alone	No	Ref	Ref	Ref	Ref
	Yes	−1.52 (−6.76, 3.72)	−1.58 (−7.26, 4.09)	−2.92 (−7.30, 1.46)	−3.62 (−9.19, 1.95)
Educational attainment	≥13 years	Ref	Ref	Ref	Ref
	≤9 years	2.63 (−4.19, 9.45)	−2.93 (−12.34, 6.48)	−0.35 (−5.96, 5.25)	−3.82 (−11.38, 3.73)
	10–12 years	3.99 (−2.43, 10.40)	−2.50 (−10.5, 5.51)	4.51 (−0.77, 9.78)	−0.22 (−6.30, 5.87)
*Covariates*					
Age	by 10 years	0.30 (−1.64, 2.23)	−0.59 (−2.01, 0.83)	−0.19 (−1.73, 1.36)	0.55 (−1.11, 2.21)
Sex	Female	Ref	Ref	Ref	Ref
	Male	−1.45 (−6.48, 3.57)	−4.36 (−9.59, 0.86)	−0.81 (−5.07, 3.44)	−0.09 (−4.82, 4.65)
Medical institution	A	Ref	Ref	Ref	Ref
	B	−2.76 (−8.84, 3.31)	4.97 (0.01, 9.92)	2.4 (−2.73, 7.54)	−1.22 (−6.62, 4.17)
Baseline PCS-8/MCS-8 score	by 1 unit	−0.52 (−0.78, −0.26)	−0.47 (−0.82, −0.12)	−0.52 (−0.72, −0.32)	−0.42 (−0.63, −0.21)

CI, confidence interval; Coef., coefficient; MCS, mental health component summary; PCS, physical health component summary.

Age, sex, healthcare institution, and baseline PCS-8 and MCS-8 scores were used to calculate multivariable-adjusted coefficients of the changes in PCS-8 and MCS-8 scores and the 95% CI of each explanatory variable.

The sensitivity analyses showed that educational attainment and baseline HRQOL scores showed slightly high correlation (eTable 1). The results of multiple linear regression excluding these variables showed similar results (eTable 2). The post-hoc power was 0.802.

## DISCUSSION

This was the first study to identify the association between the socioeconomic attributes of FLCMC applicants and changes in their HRQOL. Among patients receiving FLCMC benefits, HRQOL scores increased in those with lower income, but potentially decreased in those who lived alone. From the result concerning the intercept and the coefficient of income in the univariable regression analysis, we were able to determine that PCS-8 scores declined when participants had a monthly household equivalent income above 84,500 JPY.

Our finding concerning higher HRQOL scores in patients with lower income may be explained by changes in their patterns of consumption expenditure. Exemption of out-of-pocket payments for medical care may have enabled patients with financial difficulties to substitute the exempted money for food and other items for their well-being. Given that people in poverty have poorer health,^1^ medical expenditure may contribute to a higher proportion of their household expenses. Besides, our finding that living alone, a potential risk factor for social isolation and loneliness,^16^^,^^17^ could be associated with a decrease in both PCS-8 and MCS-8 scores may be explained by withdrawal from social activities. Although the FLCMC can provide financial support for receiving medical care and MSWs can offer a certain degree of instrumental and emotional social support,^18^ the program can be better geared to provide additional support for patients living alone. For example, a community-based intervention (eg, social prescribing) to address patients’ isolation in healthcare institutions has recently been discussed.^19^^,^^20^ This support system may have a positive effect on the HRQOL of impoverished people who live alone.

Recent Japanese studies on public assistance, which is closely related to the FLCMC, showed that living alone was associated with a higher incidence of newly diagnosed diabetes or frequent outpatient attendance.^21^^,^^22^ The Japanese public assistance program has strengthened support for self-reliance and health management to provide recipients with extra non-financial benefits.^23^^,^^24^ The FLCMC may also need to construct similar support channels for its beneficiaries by strengthening medical-welfare governance.

### Limitations

This study has several limitations. First, because we used social work data, which were not linked with personal health records or claims data, we could not consider patients’ medical conditions. In the future, the FLCMC registry will need to be merged with patients’ medical records. Second, generalizability is limited because this study used data from only two healthcare institutions in Kyoto. As the FLCMC has been implemented regionally, establishing registries of FLCMC users in each healthcare institution will be required to verify the effect of the program in Japan. Third, the sample size was small. However, based on several previous studies, our post-hoc power analysis showed desirable statistical power.

### Conclusion

The FLCMC is an emergent medical care subsidy program for impoverished patients in Japan. Our study revealed that HRQOL scores increased among the lower-income beneficiaries of the FLCMC. Living alone was potentially associated with a decrease in HRQOL scores. Through the strengthening of medical-welfare collaborations, the FLCMC might be better positioned to provide additional community-based support for patients living alone.

## SUPPLEMENTARY MATERIAL

eTable 1. Correlation coefficient between each explanatory variables and covariates

eTable 2. Adjusted coefficients and 95% confidence intervals for change in PCS-8 and MCS-8 scores by individual characteristics
