# Supplementary material for: Changes in Health-related Quality of Life Among Impoverished Persons in the Free/Low-Cost Medical Care Program in Japan: Evidence From a Prospective Cohort Study
Source: J Epidemiol. 2022 Nov 5;32(11):519–23. doi: 10.2188/jea.JE20210005 (PMC9551295; doi:10.2188/jea.JE20210005)
Supplement: Supplementary file 1 [file je-32-519-s001.pdf]

eTable 1. Correlation coefficient between each explanatory variables and covariates

	Monthly household equivalent income	Living alone	Educational attainment	Age	Sex	Baseline PCS-8 scores	Medical Institutions
Monthly household equivalent income	1.00						
Living alone	-0.11	1.00					
Educational attainment	0.29	0.16	1.00				
Age	-0.22	0.20	-0.35	1.00			
Sex	-0.09	0.09	-0.09	-0.16	1.00		
Baseline PCS-8 scores	0.00	0.11	0.41	-0.24	0.01	1.00	
Medical Institutions	-0.11	0.06	0.13	0.10	-0.21	-0.02	1.00

	Monthly household equivalent income	Living alone	Educational attainment	Age	Sex	Baseline MCS-8 scores	Medical Institutions
Monthly household equivalent income	1.00						
Living alone	-0.11	1.00					
Educational attainment	0.29	0.16	1.00				
Age	-0.22	0.20	-0.35	1.00			
Sex	-0.09	0.09	-0.09	-0.16	1.00		
Baseline MCS-8 scores	-0.12	0.21	0.10	0.01	0.07	1.00	
Medical Institutions	-0.11	0.06	0.13	0.10	-0.21	0.02	1.00

MCS, mental health component summary; PCS, physical health component summary.

eTable 2. Adjusted coefficients and 95% confidence intervals for change in PCS-8 and MCS-8 scores by individual characteristics

Character	Category	PCS<br>Adjusted Coef. (95% CI)	MCS<br>Adjusted Coef. (95% CI)
Explanatory variables			
Equivalent income	by 1,000 yen	-0.08 (-0.13, -0.02)	-0.01 (-0.08, 0.06)
Living alone	No	Ref	Ref
	Yes	-2.11 (-7.31, 3.10)	-4.87 (-10.20, 0.47)
Covariates			
Age	by 10 years	-0.08 (-1.69, 1.53)	0.36 (-1.28, 1.99)
Sex	Male	Ref	Ref
	Female	4.37 (-0.85, 9.58)	-0.09 (-5.33, 5.16)
Medical institution	A	Ref	Ref
	B	5.27 (-0.48, 9.66)	-0.34 (-7.28, 6.61)

CI, confidence interval; Coef., coefficient; MCS, mental health component summary; PCS, physical health component summary.
Educational attainment and Baseline PCS-8 and MCS-8 were removed from the analyses because of slightly high correlations. Age, sex, healthcare institution were used to calculate multivariable-adjusted coefficient of the changes in PCS-8 and MCS-8 scores and its 95% CIs of each explanatory variable.
